# Liver transcriptome and physiological analyses preliminarily revealed the adaptation mechanisms of Amur grayling (*Thymallus arcticus grubei*, Dybowski, 1869) fry for dietary lipid nutrition

**DOI:** 10.3389/fvets.2024.1369845

**Published:** 2024-04-17

**Authors:** Ze Fan, Kai Ma, Yan Wang, Liansheng Wang, Yongquan Zhang, Chenhui Li, Jiaxin Li, Di Wu, Jinnan Li, Zhengwei Li

**Affiliations:** ^1^Key Laboratory of Aquatic Animal Diseases and Immune Technology of Heilongjiang Province, Heilongjiang River Fisheries Research Institute, Chinese Academy of Fishery Sciences, Harbin, China; ^2^Tianjin Key Lab of Aqua-Ecology and Aquaculture, College of Fisheries, Tianjin Agricultural University, Tianjin, China; ^3^Supervision, Inspection and Testing Center for Fishery Environment and Aquatic Products (Harbin), Ministry of Agriculture and Rural Affairs, Harbin, China; ^4^Heilongjiang Aquatic Animal Resource Conservation Center, Harbin, China

**Keywords:** Amur grayling, dietary lipid, growth performance, liver lipid metabolism, feedback regulation mechanism

## Abstract

The Amur grayling (*Thymallus arcticus* grubei Dybowski, 1869), a species of potentially economic and research value, is renowned for its tender meat, exquisite flavor, and high nutritional contents. This study was conducted to investigate the physiological adaptation mechanisms to dietary lipids in Amur grayling fry (with average initial weight 4.64±0.03 g). This study involved a 56-day feeding trial with diets containing varying lipid levels (9.07%, 12.17%, 15.26%, 18.09%, 21.16%, and 24.07%, designated as GL1 through GL6, respectively) to explore the impact of dietary lipids on growth performance, intestinal digestion, liver antioxidative function, and transcriptomic profiles. Results showed that The group receiving 18% dietary lipid exhibited a markedly higher weight gain rate (WGR) and specific growth rate compared to other groups, alongside a reduced feed conversion ratio (FCR), except in comparison to the 15% lipid group. Activities of lipase in pancreatic secretion and amylase in stomach mucosa peaked in the 18% lipid treatment group, indicating enhanced digestive efficiency. The liver of fish in this group also showed increased activities of antioxidative enzymes and higher levels of glutathione and total antioxidative capacity, along with reduced malondialdehyde content compared to the 9% and 24% lipid treatments. Additionally, serum high-density lipoprotein cholesterol levels were highest in the 18% group. Transcriptomic analysis revealed four significant metabolic pathways affected: Cholesterol metabolism, Fat digestion and absorption, PPAR signaling, and Fatty acid degradation, involving key genes such as Lipase, Lipoprotein lipase, Fatty acid-binding protein, and Carnitine palmitoyltransferase I. These findings suggest that the liver of Amur grayling employs adaptive mechanisms to manage excessive dietary lipids. Quadratic regression analysis determined the optimal dietary lipid levels to be 16.62% and 16.52%, based on WGR and FCR, respectively. The optimal dietary lipid level for juvenile Amur grayling appears to be around 18%, as evidenced by improved growth performance, digestive function, balanced serum lipid profile, and enhanced liver antioxidative capacity. Exceeding this lipid threshold triggers both adaptive and potentially detrimental liver responses.

## Introduction

The Amur grayling (*Thymallus arcticus grubei*, Dybowski, 1869), a member of the Salmoniformes, Salmonidae, and Thymallus families, is mainly distributed in Eurasia and the tributaries of Heilongjiang River basin in China. It is a rare cold-water fish unique to the Heilongjiang River system. This species is esteemed for its tender meat, appealing taste, and high nutritional value (1). Recent declines in the wild population, primarily due to overfishing, environmental degradation, and habitat destruction, have led to the Amur grayling being classified as a vulnerable species in China (2). Despite advances in artificial breeding (3), seed cultivation (4), and germplasm identification (5) in China, comprehensive studies on the nutritional needs of Amur grayling remain unreported.

Identifying optimal dietary protein and fat levels is fundamental in the nutrition research of cold-water fish (6). Research by Gu et al. (7) indicated that the crude protein content in Amur grayling muscle is 17.93%, lower than that in rainbow trout (*Oncorhynchus mykiss*) (8), *Oncorhynchus mosou* (9), and taimen (*Hucho taimen*) (10), suggesting a relatively higher protein requirement for Amur grayling compared to lipid. The persistent escalation in prices for premium animal and plant protein sources (e.g., fishmeal and soybean meal) has increased aquaculture costs significantly (11). Leveraging the protein-sparing effect of lipids offers a viable solution to this challenge (12). Additionally, dietary lipids serve not only as a source of energy and essential fatty acids for Amur grayling but also play a role in vitamin and hormone synthesis and in the absorption of fat-soluble vitamins (13). Thus, it is critical to delineate the nutritional and physiological adaptive ranges, as well as the adaptive mechanisms, of Amur grayling to dietary lipid supplementation to support sustainable and healthy aquaculture practices.

RNA sequencing (RNA-Seq), or transcriptome sequencing, entails the sequencing of all RNA types within a cell or tissue at a given developmental stage or under specific conditions, encompassing both mRNA and non-coding RNA (14). This technology has been extensively applied across various fish species, including largemouth bass (*Micropterus salmoides*) (15) and *Brachymystax lenok* (16), to elucidate physiological regulatory mechanisms at the molecular level in response to varying dietary lipid levels. For fish, the liver is a major site where lipolysis and lipogenesis occur, which plays essential roles in the synthesis and utilization of lipids. Many key enzymes and transcriptional factors are involved in the processes of lipolysis and lipogenesis, including fatty acid desaturas 2 (FADS2), Δ6 desaturase (d6D), fatty acid elongases 2 and 5 (ELOVL2 and ELOVL5), carnitine palmitoyltransferase I (CPT I), lipoprotein lipase (LPL), fatty acid synthetase (FAS), glucose 6-phosphate dehydrogenase (G6pd), PPARs, etc. (17). Li e al. (18) found that the ability of triploid rainbow trout to synthesize polyunsaturated fatty acids (PUFAs) is weaker than that of diploids depending on comparative metabolomics analysis and quantitative PCR (qPCR) verification of the liver. Hence, studies on the response of the liver to different dietary lipid levels could help us to understand the adaptation mechanism of Amur grayling to dietary lipid nutrition.

Building on this foundation, our study focuses on the regulatory mechanisms of lipid nutrition in Amur grayling. The objective is to investigate the physiological adaptation mechanisms to dietary lipids in Amur grayling fry by examining liver and intestinal digestive physiology and hepatic antioxidant capacity and conducting comprehensive hepatic transcriptome sequencing analysis. Ultimately, this research aims to provide a theoretical framework for the development of scientifically formulated compound feeds tailored to the nutritional needs of Amur grayling.

## Materials and methods

### Ethics statement

All animal procedures employed in this study adhered to the Heilongjiang River Fisheries Research Institute’s guidelines for the care and use of laboratory animals. Ethical review and approval were obtained from the Committee for the Welfare and Ethics of Laboratory Animals at the Heilongjiang River Fisheries Research Institute (CAFS), ensuring compliance with established ethical standards.

### Experimental diet preparation

According to Cui’s (3) introduction of artificial breeding technology of Amur grayling fry, the crude protein content of the feed was 46–49%. Based on this, the protein level of the experimental diets in our study was designed to be approximately 47.5%. Fishmeal, chicken powder, cottonseed protein concentrate, and soybean protein concentrate were used as the main protein sources. Referring to a description of the range of dietary lipid requirements for cold water fish (such as rainbow trout, sturgeon, taimen, etc.) by Wang and Liu (13), the experimental diets were designed to contain six lipid levels, and the analyzed lipid concentrations were 9.07, 12.17, 15.26, 18.09, 21.16, and 24.07% (measured using the Soxhlet method) and were named as GL1, GL2, GL3, GL4, GL5, and GL6, respectively. Equal amounts of fish oil and soybean oil were used as sources of lipids, and the graded concentrations of 2.4, 3.9, 5.4, 6.9, 8.4, and 9.9% dry matter were designed to realize the increasing dietary lipid level. Corn starch is the only source of carbohydrates, the contents of which are gradually decreased with increased lipid contents. The proximate composition of experimental diets is presented in Table 1. Feed processing operations were consistent with a previous study (21). The diets were air-dried to 10% moisture at 60°C, sealed, and kept at −20°C until used.

**Table 1:** Formulation and proximate composition of the experimental diets (% dry matter).

Ingredients	Dietary lipid levels (%)					
	9 (GL1)	12 (GL2)	15 (GL3)	18 (GL4)	21 (GL5)	24 (GL6)
Chicken powder^a^	15.5	15.5	15.5	15.5	15.5	15.5
Corn starch	15	12	9	6	3	0
Fish meal^a^	42.5	42.5	42.5	42.5	42.5	42.5
Cottonseed protein concentrate^a^	9	9	9	9	9	9
Soybean protein concentrate^a^	6	6	6	6	6	6
Fish oil	2.4	3.9	5.4	6.9	8.4	9.9
Soybean oil	2.4	3.9	5.4	6.9	8.4	9.9
Vitamin^b^	0.5	0.5	0.5	0.5	0.5	0.5
Choline chloride	0.5	0.5	0.5	0.5	0.5	0.5
Monocalcium phosphate	2	2	2	2	2	2
Mineral substance^c^	0.5	0.5	0.5	0.5	0.5	0.5
Microcrystalline cellulose	3.7	3.7	3.7	3.7	3.7	3.7
Analytical composition						
Moisture^d^	10.64	10.61	10.60	10.60	10.60	10.61
Crude protein^d^	47.49	47.50	47.49	47.48	47.46	47.48
Crude lipid^d^	9.07	12.17	15.26	18.09	21.16	24.07
Crude ash^d^	8.08	8.08	8.07	8.08	8.07	8.08
Crude fibre	5.43	5.42	5.43	5.43	5.42	5.44
NFE^e^	19.29	16.32	13.33	10.33	7.32	4.32
Crude energy (kJ/g)^f^	18.11	18.78	19.45	20.12	20.79	21.46
P/E ratio (mg CP/kJ)	26.26	25.28	24.41	23.60	22.84	22.13

a Fish meal: crude protein 67.0% dry matter, crude lipid 4.85% dry matter; Chicken powder: crude protein 66.0% dry matter, crude lipid 1.30% dry matter; Cottonseed protein concentrate: crude protein 63.1% dry matter, crude lipid 1.34% dry matter; Soybean protein concentrate: crude protein 630.0 g/kg dry matter, crude lipid 5.0 g/kg dry matter. The above raw materials were purchased from Hehe Feed Co. LTD., Harbin, China.

b The vitamin premix supplied by Guangdong Hyint Biotechnology Group Co. Ltd provided the following per kg of the diet: VA 8000 IU, VC 500 mg, VD3 3000 IU, VE 60 mg, VK 3 5 mg,VB2 30 mg, VB6 15 mg, VB12 0.5 mg, choline chloride 5000 mg, nicotinic acid 175 mg, D-biotin 2.5 mg, inositol 1000 mg, folic acid 5 mg, pantothenic acid 50 mg.

c The mineral premix supplied by Guangdong Hyint Biotechnology Group Co. Ltd provided the following per kg of the diet: zinc (Zn) 25 mg, copper (Cu) 3 mg, iron (Fe) 25 mg, manganese (Mn) 15 mg, Iiodine (I) 0.6 mg, cobalt (Co) 0.1 mg, selenium (Se) 0.4 mg.

d Analytical composition analysis was conducted depending on the standard procedure of the AOAC (2005) (19). Moisture was determined by oven drying to a constant weight at 105°C. Crude protein was analyzed using the Kjeldahl method after acid digestion. Crude lipids were determined by using the other extraction method with a Soxtec system. Crude ash was determined after combustion to a constant weight at 550°C.

e NFE = nitrogen-free extract. Estimated by the formula 1,000 − (water + crude protein + crude fiber + ash + crude lipid).

f Calculated using the mean values for carbohydrates (17.2 kJ/g), proteins (23.6 kJ/g), and lipids (39.5 kJ/g) according to the NRC (2011) (20).

### Fish rearing and experimental conditions

The Amur grayling fry was provided by the Bohai Cold-Water Fish Experimental Station of Heilongjiang River Fisheries Research Institute, Chinese Academy of Fishery Sciences. Amur grayling fry were reared in 120 L recirculating aquaculture system (RAS) aquaria (38 cm × 39 cm × 80 cm) for 2 weeks before the feeding trial to adapt to the environment. A total of 450 Amur grayling fry (initial body weight 4.64 ± 0.03 g) were randomly distributed into 18 aquaria, with 25 Amur grayling fry in each aquarium. Each aquarium was randomly assigned to one of the six experimental diets, resulting in three replicates for each diet. They were artificially fed 2–3% of body mass three times daily (8:00, 13:00, and 17:00 h) based on the description of Cui (3) and Feng et al. (22) and re-weighted to adjust their daily feeding amount at fortnightly intervals. During the 8-week breeding trial, approximately one-third of water was exchanged to maintain the favorable water quality indices every third day. The water quality parameters during the experiment were as follows: water temperature was 13 ± 0.5°C, total ammonia was approximately 0.20 mg/L, and dissolved oxygen ranged from 5.8 to 6.2 mg/L.

### Sample collection

Amur grayling were weighed and counted after the 8-week breeding trial to calculate weight gain rate (WGR), specific growth rate (SGR), feed conversion rate (FCR), and survival rate. Depending on the operation procedure described by Feng et al. (22) and Zhang et al. (23), experimental fish were sacrificed on ice using 100 mg/L of MS-222 (tricaine methanesulfonate; Sigma, St. Louis, MO, United States) and dissected immediately to collect the tissue samples. MS-222 (Sigma Chemical E10521, St Louis, MO, United States) used in the experiment was solubilized in deionized water and buffered with sodium bicarbonate at a ratio of 1:1 (sodium bicarbonate:tricaine methanesulfonate powder), providing a solution with a final MS-222 concentration of 10 mg/mL (pH 7.0) from which aliquots were taken and used in anesthetization for fish (24). The mixed liver and intestine samples from three Amur grayling in each replicate were taken for enzyme activity analysis. Additionally, the mixed liver samples from the other three Amur grayling in each replicate were quickly removed and put into a 1.5-mL centrifuge tube, which was then immediately frozen with liquid nitrogen to prevent RNA degradation and stored at −80°C for gene expression analysis. Moreover, according to the growth performance, the obtained liver samples from three other Amur grayling in GL1, GL3, and G6 groups were used for transcriptome analysis.

### Detection of enzyme activity

Each of the liver, stomach, and intestine in different groups was accurately weighted (0.2 g) and homogenized in 0.9 mL of 0.9% saline with a low-temperature homogenizer and high-speed refrigerated centrifuge according to our previous study (21).

An assay kit produced by Nanjing Jiancheng Bioengineering Institute was used to assess the digestive ability of the stomach and intestine, including lipase (Cat. No. A054-2-1) and amylase (Cat. No. C016-1-1). Another assay kit produced by Nanjing Jiancheng Bioengineering Institute was used to assess the antioxidant ability in liver and intestine by measuring the activities of catalase (Cat. No. A007-2-1) and peroxidase (POD, Cat. No. A084-3-1), as well as the contents of glutathione (GSH, Cat. No. A006-2-1), malondialdehyde (MDA), and total antioxidant capacity (T-AOC, Cat. No. A015-2-1). The double antibody sandwich method was used for the activities of protease in the stomach and intestine, as well as superoxide dismutase (SOD) in the liver and intestine, using an enzyme-linked immunosorbent assay (ELISA) kit produced by Shanghai Enzyme-Linked Biotechnology Co., LTD, as described in our previous study (21). The activities of glutamic oxalacetic transaminase (GOT) and glutamic-pyruvic transaminase (GPT) in the liver were detected using commercial kits from Nanjing Jiancheng Bioengineering Institute (Corresponding commodity serial numbers: Cat. No C0013-2-1, C010-2-1). The protein concentration of the tissue homogenate determined by coomassie blue staining was used to calculate relevant enzyme activity indicators.

### Detection of serum biochemical parameters

The contents of triglyceride (TG), total cholesterol (TCHO), high-density lipoprotein cholesterol (HDL-C), and low-density lipoprotein cholesterol (LDL-C) in the serum were detected using commercial kits from Nanjing Jiancheng Bioengineering Institute (Corresponding commodity serial numbers: Cat. No C009-2-1, A110-1-1, A111-1-1, A112-1-1, A113-1-1).

### Transcriptome profiling analysis of liver

Liver total RNA from Amur grayling was extracted with an RNAiso Plus Kit (Cat. No. 9109, TaKaRa, Co. Ltd., Dalian, China). The RNA integrity and quantity were assessed using an Agilent 2,100 Bioanalyzer (Agilent, Santa Clara, CA). Library construction and sequencing were carried out by Shanghai Majorbio Bio-pharm Biotechnology Co., Ltd. (Shanghai, China). Three samples were collected in each library with an equal amount of purified RNA. Each library was sequenced using 2 × 125 bp pair-end (PE) mode in the Illumina HiSeq 6,000 sequencing platform.

Raw paired-end reads were trimmed using SeqPrep software^1^ and quality controlled using Sickle software^2^ with default parameters. Clean reads were then used for *de novo* assembly with Trinity software.^3^ The web program BLAST was used to annotate unigenes with the Nt database to obtain complete gene function information. All the assembled sequences were assigned to the NCBI protein non-redundant (NR), the Swiss-Prot protein database, Clusters of Orthologous Groups of proteins (COG), Gene Ontology (GO), and Kyoto Encyclopedia of Genes and Genomes (KEGG) databases using BLASTX (E-value cutoff of 1e-5) for functional annotations (25).

Bowtie2 software in RSEM was used to compare the clean reads of each sample with the transcriptome spliced by Trinity to obtain the number of read counts on each gene. Then, the read count data were standardized to analyze the gene expression level. DEG-seq provided a statistical method for determining differentially expressed genes (DEGs), and DEG-seq was used for differential analysis. To reduce the number of false positives, the screening threshold was set to *q*-value < 0.01 and |log2 Fold Change| > 2.

### Data processing and analysis

To assess the growth performance of the common carp, the weight gain rate (WGR), specific growth rate (SGR), protein efficiency ratio (PER), and feed conversion ratio (FCR) were computed in terms of the following formula:

$$WGR\;\left(\%\right)=100\times \left[\left(\mathrm{final}\;\mathrm{weight}-\mathrm{initial}\;\mathrm{weight}\right)\right]/\mathrm{initial}\;\mathrm{weight}\text{;}$$

$$SGR\;\left(\%/d\right)=100\times \left[\left(Ln\;\mathrm{final}\;\mathrm{weight}-Ln\;\mathrm{initial}\;\mathrm{weight}\right)/56\;\mathrm{days}\right]\text{;}$$

$$\mathrm{PER}\;\left(\%\right)=100\times \left[\begin{array}{l} \left(\mathrm{final}\;\mathrm{weight}-\mathrm{initial}\;\mathrm{weight}\right)/\\ \left(\mathrm{total}\;\mathrm{feed}\;\mathrm{given}\times \mathrm{content}\;\mathrm{of}\;\mathrm{dietary}\;\mathrm{protein}\right) \end{array}\right]\text{;}$$

$$FCR=\left[100\times \mathrm{total}\;\mathrm{feed}\;\mathrm{given}/\mathrm{total}\;\mathrm{wet}\;\mathrm{weight}\;\mathrm{gain}\right]\text{;}$$

The sum of feed input of 2–3% body weight for 56 days per aquarium was deemed as total feed giver, which was used for calculating the FCR and PER. Data analyses were carried out using SPSS 23.0 software (SPSS Inc., Chicago, IL, United States). The results were subjected to a one-way analysis of variance (ANOVA), followed by Duncan’s test to delineate significance among fish groups. Moreover, quadratic regression analyses were applied to determine the optimum lipid level based on WGR and FCR according to their changing trend (26). Experimental results were presented as mean values ± standard deviations. Statistically significant levels were assigned at *p* < 0.05 unless otherwise stated.

## Results and analysis

### Growth parameters and feed utilization

**Figure 1:**
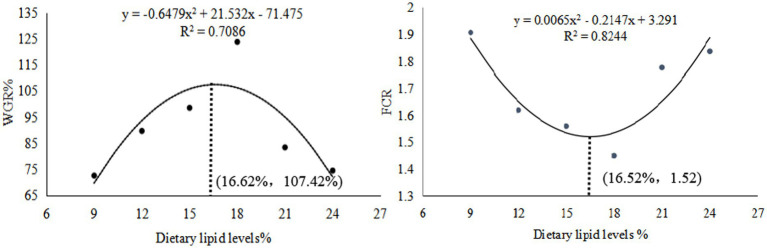
Quadratic regression relationship of WGR and FCR to dietary lipid levels.

The weight gain ratio (WGR), specific growth rate (SGR), and protein efficiency ratio (PER) exhibited an initial increase followed by a decrease, peaking in the GL4 group. Specifically, the Amur grayling in the 18% lipid group demonstrated a significantly higher WGR and SGR than other groups (*p* < 0.05). Conversely, the feed conversion ratio (FCR) displayed an inverse trend, being significantly lower in the GL4 group compared to all others except the GL3 group (*p* < 0.05) (Table 2). Figure 1 presents the quadratic regression analysis. Using WGR as the metric, the quadratic regression equation was y1 = −0.6479x^2^ + 21.532x−71.475 (R^2^ = 0.7086), indicating an optimal dietary lipid level of 16.62%. Similarly, based on FCR, the quadratic regression equation was y^2^ = 0.0065x^2^–0.2147x + 3.291 (R^2^ = 0.8244), suggesting an optimal lipid level of 16.52%.

**Table 2:** Growth performance and feed utilization of Amur grayling fed the experimental diets for 56 days.^a^

Groups	IBW/g	FBW/g	WGR/%	SGR/ (%/d)	FCR
GL1	4.64 ± 0.03	8.01 ± 0.20^d^	72.61 ± 3.41^d^	1.30 ± 0.05^d^	1.91 ± 0.10^a^
GL2	4.65 ± 0.01	8.82 ± 0.09^c^	89.67 ± 1.39^c^	1.52 ± 0.02^c^	1.62 ± 0.02^b^
GL3	4.66 ± 0.02	9.25 ± 0.08^b^	98.51 ± 2.48^b^	1.63 ± 0.03^b^	1.56 ± 0.04^bc^
GL4	4.65 ± 0.01	10.41 ± 0.13^a^	123.75 ± 2.24^a^	1.92 ± 0.02^a^	1.45 ± 0.02^c^
GL5	4.67 ± 0.00	8.57 ± 0.04^c^	83.40 ± 0.68^c^	1.44 ± 0.01^c^	1.78 ± 0.01^a^
GL6	4.67 ± 0.00	8.15 ± 0.027^d^	74.52 ± 0.49^d^	1.33 ± 0.01^d^	1.84 ± 0.02^a^

a IBW, initial body weight; FBW, final body weight; WGR, weight gain rate; SGR, specific growth rate; PER, protein efficiency ratio; FCR, Feed conversion rate. Values were presented as mean ± SD. Values in the same row with different superscripts were significantly different (*p* < 0.05) (the same as in the following tables).

### Digestive enzyme activity in the stomach

Specifically, the activities of lipase in pancreatic secretion (LPS) and amylase in stomach mucosa (AMS) followed a pattern of initial increase followed by a decrease, peaking in the GL4 group. Notably, the AMS activity in the 18% group (GL4) exhibited significant increases compared to the GL1, GL5, and GL6 groups (*p* < 0.05) (Table 3).

**Table 3:** Digestive enzyme activity in the stomach of Amur grayling fed the experimental diets for 56 days.

Groups	Protease (pg/mL)	Lipase (U/gprot)	Amylase (U/mgprot)
GL1	371.17 ± 22.99	4.62 ± 1.49	0.21 ± 0.02^b^
GL2	337.00 ± 11.39	8.27 ± 1.19	0.28 ± 0.03^ab^
GL3	316.17 ± 59.04	7.19 ± 2.80	0.28 ± 0.03^ab^
GL4	312.00 ± 61.32	11.25 ± 2.22	0.32 ± 0.01^a^
GL5	343.67 ± 36.36	6.11 ± 1.23	0.24 ± 0.03^b^
GL6	331.17 ± 28.91	5.79 ± 2.97	0.24 ± 0.004^b^

### Digestive enzyme activity in the intestine

Data presented in Table 4 show that the activities of intestinal protease, lipase in pancreatic secretion (LPS), and amylase (AMS) in stomach mucosa demonstrated a trend of initial increase and subsequent decrease correlating with rising dietary lipid levels. All three enzymes reached their peak activities in the GL4 group. Specifically, the GL4 group exhibited significantly higher protease activity compared to the GL1, GL2, and GL6 groups and markedly higher AMS activities relative to all groups except GL3 (*p* < 0.05).

**Table 4:** Digestive enzyme activities in the intestine of Amur grayling fed the experimental diets for 56 days.

Groups	Protease (pg/mL)	Lipase (U/gprot)	Amylase (U/mgprot)
GL1	170.33 ± 50.28^bc^	14.31 ± 7.26	0.79 ± 0.02^d^
GL2	172.5 ± 75.34^bc^	19.34 ± 10.67	1.25 ± 0.16^c^
GL3	314.83 ± 35.08^ab^	22.32 ± 7.91	1.80 ± 0.04^ab^
GL4	331.17 ± 17.07^a^	27.12 ± 10.77	1.86 ± 0.05^a^
GL5	294.50 ± 52.97^abc^	4.78 ± 0.87	1.56 ± 0.14^b^
GL6	159.50 ± 22.91^bc^	4.90 ± 2.03	0.86 ± 0.04^d^

### Got and GPT activities related to liver function

Table 5 illustrates that the activities of glutamic-oxaloacetic transaminase (GOT) and glutamic-pyruvic transaminase (GPT) in the liver initially increased and then decreased with an increase in dietary lipid level. Notably, the GL4 group exhibited significantly higher GOT activity than all other groups, except for the GL3 group, and demonstrated higher GPT activity than all other groups (*p* < 0.05).

**Table 5:** GOT and GPT activities related to the liver function of Amur grayling fed the experimental diets for 56 days.

Groups	GOT (U/gprot)	GPT (U/gprot)
GL1	4.93 ± 0.77^b^	2.58 ± 0.25^c^
GL2	6.80 ± 0.60^a^	2.92 ± 0.13^bc^
GL3	7.50 ± 0.41^a^	3.35 ± 0.20^b^
GL4	8.37 ± 0.31^a^	4.02 ± 0.14^a^
GL5	3.58 ± 0.55^bc^	2.95 ± 0.28^bc^
GL6	3.32 ± 0.24^c^	1.83 ± 0.08^d^

### Antioxidant parameters in the liver

Table 6 demonstrates the variation in antioxidant parameters in the liver, indicating that the activities of superoxide dismutase (SOD), catalase (CAT), peroxidase (POD), glutathione (GSH), and total antioxidant capacity (T-AOC) initially increased and then decreased, reaching peak values in the GL4 group. Specifically, the GL4 group exhibited significantly higher SOD activity and GSH content than all groups except GL3, increased CAT activity relative to the GL1 and GL6 groups, and elevated T-AOC content in comparison to the GL1 and GL2 groups (*p* < 0.05). Additionally, this group showed notably lower malondialdehyde (MDA) content than the other groups (*p* < 0.05).

**Table 6:** Antioxidant parameters in the liver of Amur grayling fed the experimental diets for 56 days.

Groups	SOD (IU/mL)	CAT (U/mgprot)	POD (U/mgprot)	GSH (μmol/gprot)	T-AOC (mmol)	MDA (nmol/mgprot)
GL1	7.06 ± 0.16^cd^	81.77 ± 6.36^b^	11.87 ± 0.33^bc^	2.70 ± 0.37^cd^	0.18 ± 0.04^b^	4.72 ± 0.80^b^
GL2	8.29 ± 0.58^b^	127.11 ± 2.33^a^	12.63 ± 0.31^ab^	4.48 ± 0.73^bc^	0.19 ± 0.07^b^	4.01 ± 0.82^b^
GL3	8.48 ± 0.24^ab^	127.46 ± 5.56^a^	13.65 ± 0.36^a^	6.12 ± 0.77^ab^	0.34 ± 0.03^a^	5.14 ± 0.37^b^
GL4	9.31 ± 0.08^a^	130.72 ± 2.47^a^	14.01 ± 0.46^a^	6.89 ± 1.15^a^	0.35 ± 0.02^a^	1.88 ± 0.25^c^
GL5	7.88 ± 0.39^bc^	111.15 ± 4.87^a^	11.72 ± 0.78^bc^	1.92 ± 0.44^d^	0.25 ± 0.05^ab^	8.95 ± 1.04^a^
GL6	6.58 ± 0.10^d^	59.94 ± 11.78^c^	10.53 ± 0.29^c^	2.92 ± 0.42^cd^	0.23 ± 0.03^ab^	10.84 ± 0.41^a^

### Lipid composition in serum

High-density lipoprotein cholesterol (HDL-C) levels initially decreased and then increased, reaching their highest values in the GL4 group. This group exhibited significantly higher HDL-C content compared to the other groups and elevated triglyceride (TG) content, except when compared to the GL2 and GL3 groups (*p* < 0.05). Conversely, low-density lipoprotein cholesterol (LDL-C), total cholesterol (TCHO), and TG displayed the reverse trend. Notably, the GL4 group had significantly lower LDL-C content than other groups, except GL2 and GL3, and lower T-CHO and TG contents compared to all groups except GL1 (*p* < 0.05) (Table 7).

**Table 7:** Lipid composition in the serum of Amur grayling fed the experimental diets for 56 days.

Groups	HDL-C (mmol/gprot)	LDL-C (mmol/gprot)	TCHO (mmol/gprot)	TG (mmol/gprot)
GL1	0.21 ± 0.03^c^	0.17 ± 0.01^b^	0.04 ± 0.01^d^	0.18 ± 0.04^cd^
GL2	0.32 ± 0.02^b^	0.06 ± 0.01^c^	0.21 ± 0.01^b^	0.31 ± 0.01^a^
GL3	0.34 ± 0.02^b^	0.05 ± 0.01^c^	0.14 ± 0.02^c^	0.24 ± 0.01^bc^
GL4	0.43 ± 0.02^a^	0.04 ± 0.01^c^	0.04 ± 0.02^d^	0.16 ± 0.02^d^
GL5	0.32 ± 0.03^b^	0.13 ± 0.04^b^	0.24 ± 0.01^ab^	0.28 ± 0.02^ab^
GL6	0.24 ± 0.01^c^	0.26 ± 0.01^a^	0.26 ± 0.01^a^	0.32 ± 0.01^a^

Overall, the optimal dietary lipid level for Amur grayling is identified as 18%. Levels exceeding 18% or as low as 9% significantly impede the growth, digestibility, and antioxidant performance of Amur grayling. Based on these findings, the 9% (GL1), 18% (GL4), and 24% (GL6) groups were selected for further liver transcriptome response analysis.

### Transcriptome profiling analysis of liver

#### Illumina sequencing and *de novo* assembly

Transcriptome sequencing yielded a total of 58.55 Gb of clean data, with each sample contributing approximately 6.25 Gb. The percentage of Q30 bases, an indicator of sequencing quality, exceeded 94.17% (Table 8). From the assembled clean data, a total of 81,674 unigenes were identified, among which 12,302 unigenes were over 1 kb in length (Table 9).

**Table 8:** Evaluation statistics of sample sequencing data.

Sample	Raw read number	Clean base number	GC content (%)	≥Q30 (%)
GL1_1	43,472,136	6,436,612,209	48.68	95
GL1_2	45,025,046	6,647,305,531	47.03	94.87
GL1_3	47,716,218	7,048,577,866	46.98	94.86
GL4_1	43,574,418	6,388,752,474	47.41	94.48
GL4_2	42,341,906	6,247,024,466	46.38	94.71
GL4_3	44,190,092	6,480,339,914	45.31	94.17
GL6_1	43,732,194	6,474,362,073	47.07	94.99
GL6_2	43,594,460	6,399,443,169	47.4	94.36
GL6_3	43,713,016	6,428,661,501	47.51	94.38

Raw read number, Total number of pair-end reads in clean data; Clean base number, Total bases of clean data; GC content, percentage of G and C bases in clean data to total bases; % ≥ Q30, The percentage of bases with a quality value greater than or equal to 30; GL1_1, GL1_2, and GL1_3 represent liver samples of the GL1 (9% lipid) group, respectively; GL4_1, GL4_2, and GL4_3 represent liver samples of the GL4 (18% lipid) group, respectively; GL6_1, GL6_2, and GL6_3 represent liver samples of the GL6 (24% lipid) group, respectively.

**Table 9:** The result statistics of unigene and transcript.

Length range	Unigene	Transcript
200 ~ 500	53,957 (66%)	62,200 (61%)
501 ~ 1,000	15,415 (19%)	21,519 (21%)
1,001 ~ 1,500	6,553 (8%)	9,911 (10%)
1,501 ~ 2000	3,158 (4%)	4,819 (5%)
2001 ~ 2,500	1,417 (2%)	2,147 (2%)
2,500+	1,174 (1%)	1880 (1%)
Total number	81,674	102,476
Total length	47,199,207	65,224,569
Average length (bp)	577.9	636.49
N50 length (bp)	819	943

Length range: the different length intervals of unigene; the number in the table indicates the number of unigene in the corresponding interval and the percentage in parentheses indicates the proportion of unigene in the corresponding length interval; Total number: indicates the total number of unigenes assembled; Total length: indicates the total length of the assembled unigene; N50 Length: indicates the length of unigene’s N50; Mean length: indicates the average length of unigene.

#### Functional annotation and classification

**Figure 2:**
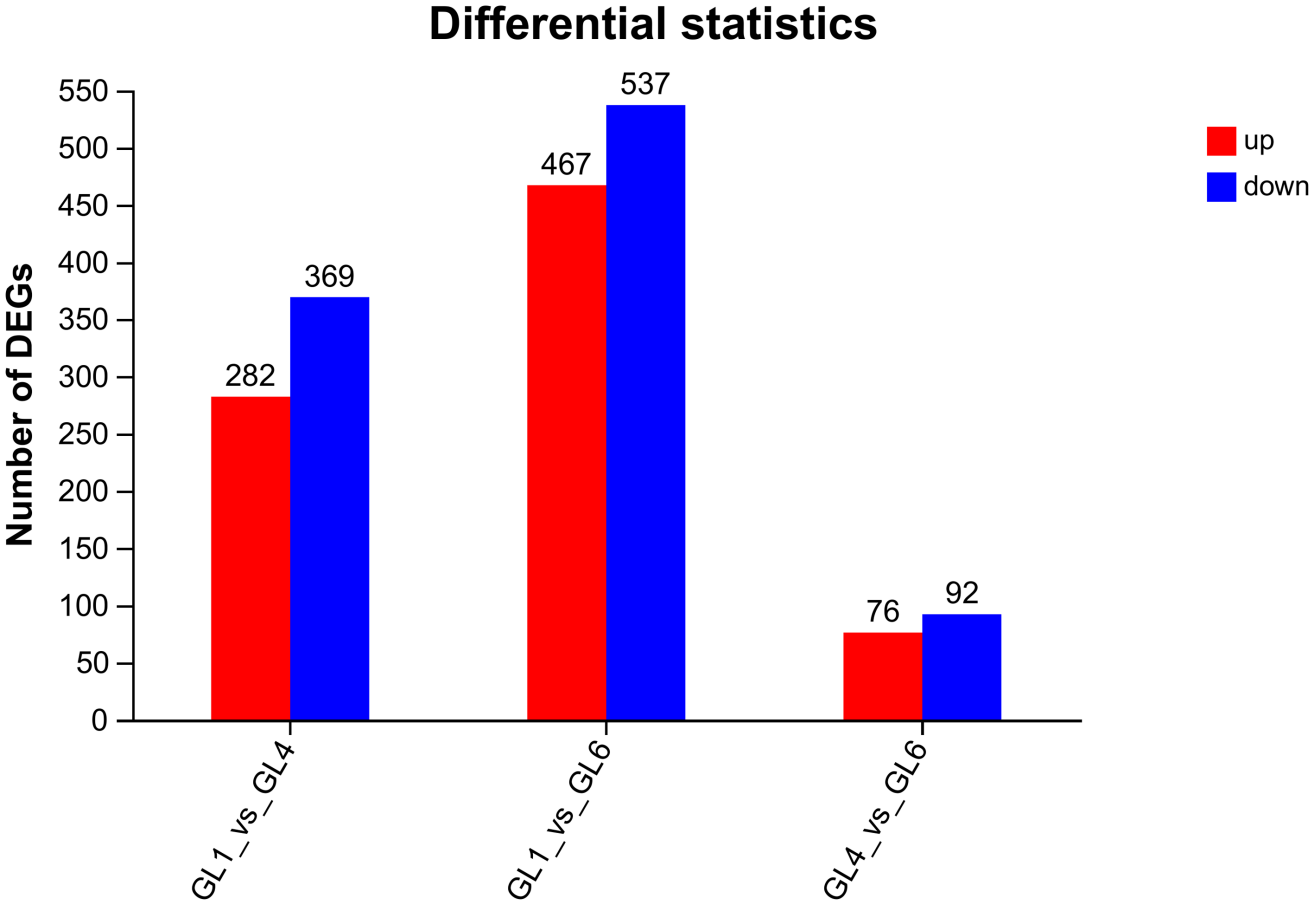
Gene ontology classification of assembled unigenes.

Functional annotation of the 80,248 unigenes was conducted using various databases, including NR, Swiss-Prot, Pfam, eggNOG, GO, and KEGG (Table 10). Of these, 29,284 unigenes (representing 36.5% of the total) were annotated in the Gene Ontology (GO) database, encompassing three main categories: biological process, cellular component, and molecular function (Figure 2). Within the biological process category, the predominant sub-categories were cellular processes, metabolic processes, and biological regulation. The cellular component category was primarily characterized by sub-categories such as cell part, membrane, and organelle. In the molecular function category, binding and catalytic activities emerged as the principal sub-categories.

**Table 10:** The annotation statistics of unigene and transcript.

	Exp_Unigene number (percent)	Exp_Transcript number (percent)
GO	29,284 (0.3649)	38,909 (0.3866)
KEGG	29,466 (0.3672)	38,763 (0.3852)
eggNOG	31,983 (0.3986)	42,793 (0.4252)
NR	40,495 (0.5046)	53,580 (0.5324)
Swiss-Prot	26,685 (0.3325)	35,809 (0.3558)
Pfam	19,617 (0.2445)	26,854 (0.2668)
Total_anno	42,880 (0.5343)	56,470 (0.5611)
Total	80,248 (1)	100,640 (1)

#### Identification of DEGs, KEGG enrichment, and function analysis

**Figure 3:**
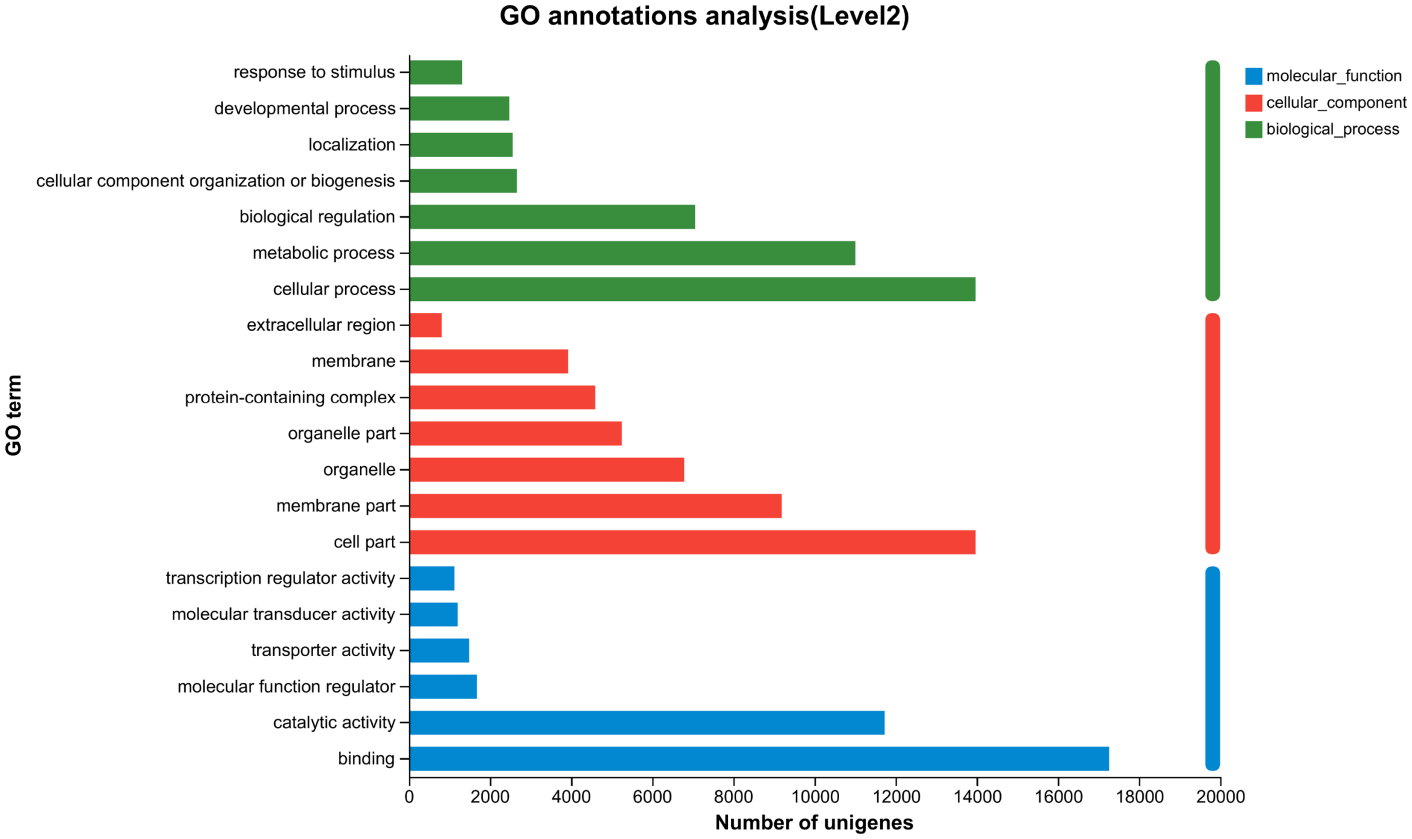
Numbers of DEGs in GL1 vs. GL4, GL1 vs. GL6, and GL4 vs. GL6 comparisons. Red and blue indicate upregulated and downregulated DEGs, respectively.

The GL1 group was designed as the matched group to detect the molecular mechanisms of liver function caused by increasing dietary lipid levels. Based on the threshold of |log2(Fold change)| > 1 and FDR < 0.05, a total of 678, 1,004, and 168 DEGs were identified in the GL1 vs. GL4, GL1 vs. GL6, and GL4 vs. GL6 comparisons, respectively (Figure 3). Moreover, Figure 3 revealed that an increment in downregulated genes was observed with increasing dietary lipid levels.

**Figure 4:**
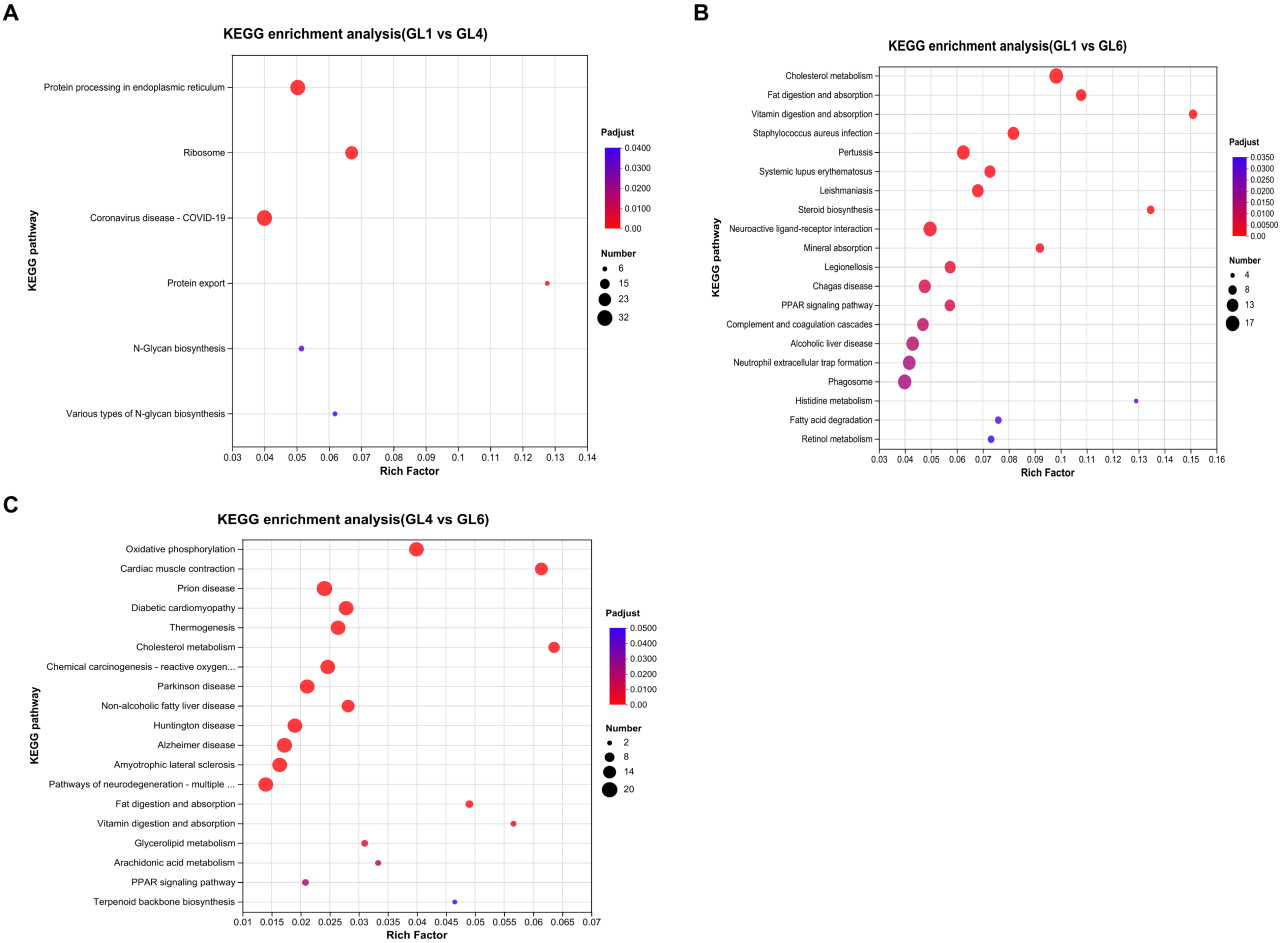
The top 20 enriched KEGG pathways in GL1 vs. GL4 **(A)**, GL1 vs. GL6 **(B)**, and GL4 vs. GL6 **(C)** comparisons. The x-axis shows the value of the gene ratio (number of DEGs enriched in the pathway/number of all genes in the annotation gene set) for each pathway. The y-axis indicates the specific KEGG pathway. The gene numbers and ranges of the Q-values are represented by the size and color of the dots, respectively.

The top 20 pathways of differentially expressed genes (DEGs), ranked by Q-value based on KEGG enrichment analyses, are displayed in Figures 4A–C. Notably, in the GL1 vs. GL4 comparison, the most significant pathways were protein processing in the endoplasmic reticulum, ribosome, and protein. Additionally, several pathways linked to lipid metabolism were prominently enriched in GL1 vs. GL6 and GL4 vs. GL6 comparisons. These include cholesterol metabolism, fat digestion and absorption, and PPAR signaling pathways. Steroid biosynthesis and fatty acid degradation pathways were particularly noted in GL1 vs. GL6, while non-alcoholic fatty liver disease was significant in GL4 vs. GL6.

**Figure 5:**
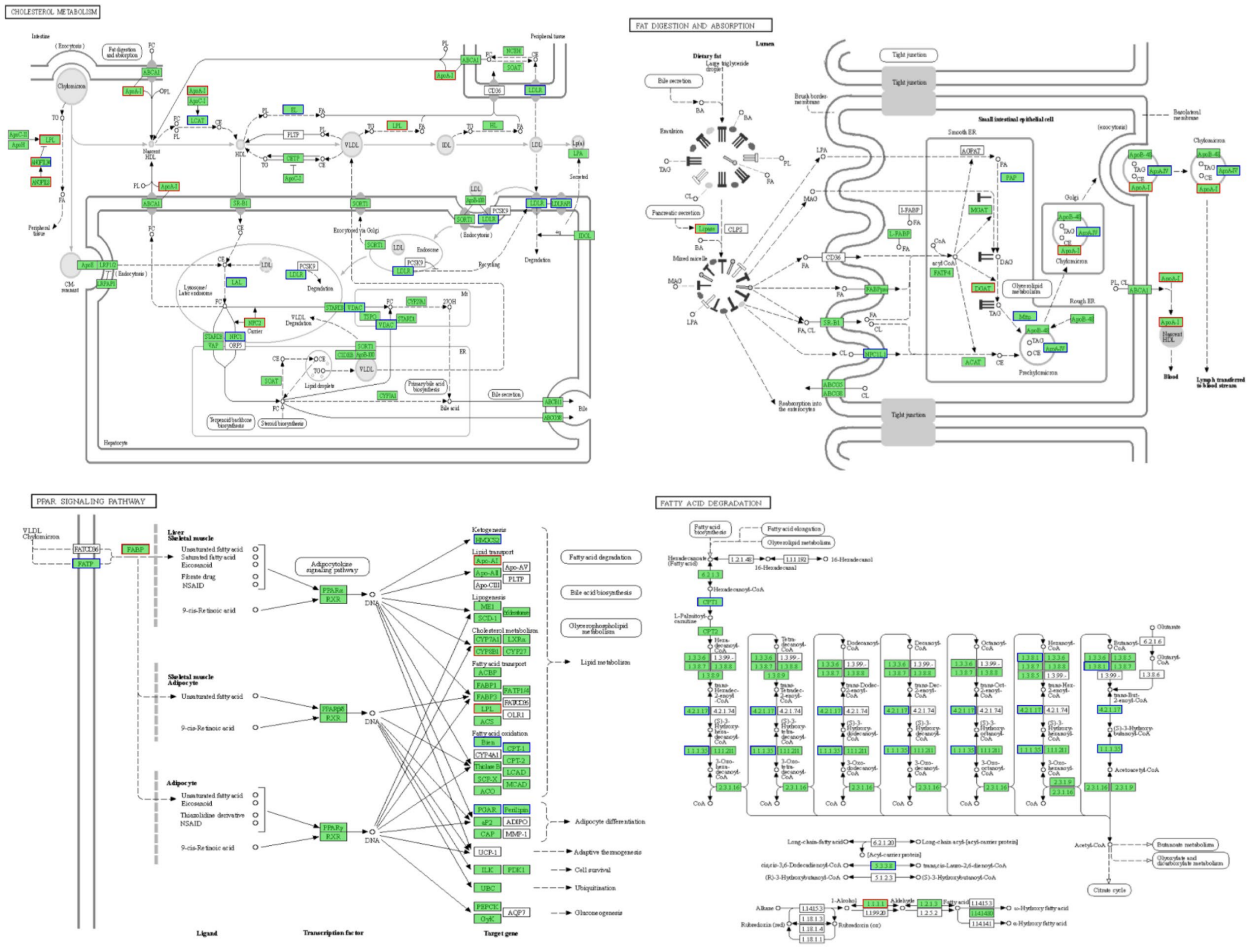
Four enriched KEGG pathways in the GL1 vs. GL6 and GL4 vs. GL6 comparisons and their involved genes.

**Figure 6:**
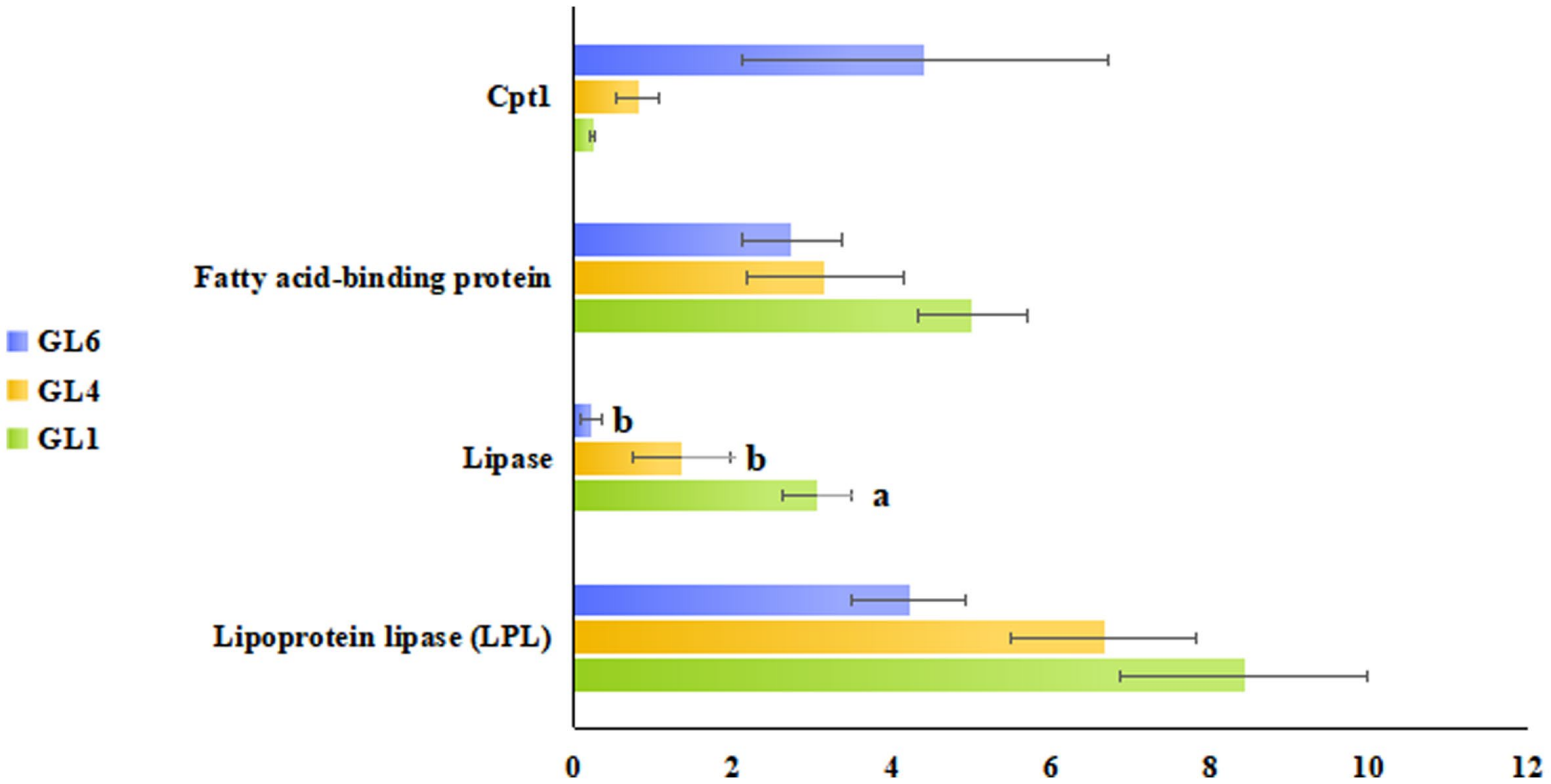
RNA-seq identification of lipoprotein lipase (LPL) in the cholesterol metabolism pathway, lipase in fat digestion and absorption pathway, fatty acid-binding protein in the PPAR signaling pathway, and Cpt1 in the fatty acid degradation pathway. Data were expressed as mean ± standard error (means ± SD) with three biological replicates.

Figure 5 illustrates these enriched pathways and their key DEGs for clearer understanding. RNA-seq analysis revealed that lipoprotein lipase (LPL) in the cholesterol metabolism pathway, lipase in the fat digestion and absorption pathway, and fatty acid-binding protein in the PPAR signaling pathway were downregulated with increased dietary lipid levels, with lipase expression being significantly higher in the GL1 group than in the GL6 group (*p* < 0.05). In contrast, carnitine palmitoyltransferase I (CPT1) in the fatty acid degradation pathway was upregulated with increased dietary lipids, though no significant differences were observed among the three groups (Figure 6). The potential functional roles of these DEGs in lipid metabolism in Amur grayling will be discussed in detail.

## Discussion

Compared to warm-water fish, cold-water fish exhibit a superior ability to utilize dietary lipids. For instance, salmon and trout can thrive with dietary lipid levels as high as 35%, while sturgeon optimal lipid levels range between 5 and 12% (13). Our study reveals that Amur grayling shows optimal performance with 18% dietary lipid, indicating efficient lipid utilization characteristic of cold-water fish. We observed that dietary lipid levels ranging from 12 to 21% positively influenced the WGR, SGR, and FCR of Amur grayling, with 18% proving to be the most effective. This pattern aligns with the study of common carp (*Cyprinus carpio*), suggesting that growth performance in fish presented the trend of initially increasing and then decreasing as dietary lipid levels rise when the designed lipid gradient is reasonable and differences between groups are adequately distinct (27). Within an appropriate physiological range, dietary lipids can significantly enhance growth, but beyond this range, they may become unsuitable and exert inhibitory effects. However, findings on lipid nutritional requirements in cold-water fish are not universally consistent. As Liu et al. discovered during their research, rainbow trout (*Oncorhynchus mykiss*) in cage culture with flowing water showed a positive correlation between growth parameters and increasing dietary lipid levels, identifying a minimum suitable lipid requirement of 26.06% (28). This suggests metabolic differences in lipid processing among various cold-water fish species, underscoring the need for further research to understand these variations.

Digestive capacity and nutrient absorption efficiency are key determinants of feed utilization and fish growth. Among digestive enzymes, protease, lipase, and amylase are critical for these processes (29). Enzyme activity is a crucial indicator of the digestive system’s physiological function, influencing nutrient digestion and absorption and ultimately affecting growth, development, and reproduction in fish (21). The Amur grayling possesses a comprehensive digestive system, including the esophagus, pyloric cecum, stomach, and intestine. Zhao and Li (29) observed that wild Amur grayling have higher trypsin and amylase activities in the intestine and pyloric cecum and elevated lipase activities in the pyloric cecum and stomach, which is consistent with their carnivorous diet. Our study revealed both similarities and notable differences in digestive enzyme activities between the stomach and intestine in response to increasing dietary lipid levels. The activities of lipase in pancreatic secretion and amylase in stomach mucosa peaked at 18% lipid treatment in both organs. Interestingly, we noted a divergence in the activity patterns of stomach proteases, which were restrained with higher lipid levels, and intestinal proteases, peaking at 18% lipid treatment. These findings suggest a gradual increase in the activities of digestive enzymes related to energy substances with higher dietary lipid content in Amur grayling fry. Importantly, the increased activity of these enzymes appears to enhance intestinal protease activity, reinforcing the protein-sparing effect of lipids. Similar observations have been reported in studies on paddlefish (*Polyodon spathula*) (30), Pengze crucian carp (*Carassius auratus* var. Pengze) (31), and common carp (27).

GPT and GOT existing in liver cells are important indicators to reflect whether the liver function is normal, as well as important enzymes to reflect the amino acid metabolism (32). When liver cells are damaged by foreign substances, a large amount of GPT and GOT penetrates the blood due to the increasing permeability of the cell membrane, resulting in increased activity of GPT and GOT in the blood (33). In the present research, Amur grayling fry in the 18% dietary lipid treatment gained higher GOT and GPT activities, indicating that the corresponding serum of this treatment has lower GOT and GPT activities. This result may indicate that under the conditions of appropriate lipid levels, the liver function of Amur grayling could not be damaged but could be improved, which was in line with the study on paddlefish (30) and *Procypris merus* (34).

High lipid levels typically reduce antioxidant capacity in fish, as observed in large yellow croaker (*Larimichthys crocea*) (35) and rainbow trout (36), characterized by disruptions in both enzymatic and non-enzymatic antioxidant systems responding to reactive oxygen species (ROS) generated during nutrient metabolism (37). Studies on largemouth bass (*Micropterus salmoides*) (38) and GIFT tilapia (*Oreochromis niloticus*) (39) found that the optimal lipid levels (11.05 and 9.35%) led to increases in SOD and GSH-Px activities and a decrease in MDA content in the liver. Analogously, our results showed that at 18% dietary lipid, the liver exhibited increased activities of SOD, CAT, POD, GSH, and T-AOC content and decreased MDA content compared to the 9 and 24% treatments. This indicates that an optimal lipid diet boosts the fish’s antioxidant capacity, facilitating a balance between ROS production and elimination, thereby mitigating oxidative stress. Hence, it can be hypothesized that the dietary lipid tolerance threshold for Amur grayling fry, based on liver antioxidant capacity, is approximately 18%.

Blood lipids play essential roles in maintaining body temperature, transporting foreign substances, preserving acid–base balance, participating in cell metabolism, nourishing cells, and supporting immunity. Serum TCHO and TG are indicators of fish nutritional status, with elevated levels reflecting active endogenous lipid transport as a response to dietary lipid intake (40). HDL acts as a cholesterol transporter in the blood, forming HDL-C upon cholesterol binding, while low-density lipoprotein (LDL) performs the opposite function, forming LDL-C (41). In our study, serum LPL-C, TCHO, and TG content significantly decreased with dietary lipid levels from 12 to 18%, in contrast to other treatments. Conversely, serum HDL-C peaked at the 18% dietary lipid level. The above results indicated that a more active endogenous lipid transport would be triggered in response to the appropriate dietary lipid level, which is similar to previous studies of Atlantic salmon (*Salmo salar*, L.) (42) and *Acipenser gueldenstaedti* (43). Additionally, Our findings suggest that in Amur grayling, excessive dietary lipids (beyond 18%) impair liver function, reducing the capacity to synthesize and secrete proteins necessary for cholesterol transport. This results in increased serum TC content and stable or declining HDL-C. A similar result was observed in the study on yellow drum (*Nibea albiflora*) (44). The specific underlying mechanisms warrant further investigation.

To elucidate the lipid metabolism regulatory mechanisms in Amur grayling, transcriptome sequencing was performed on liver samples from the 9, 18, and 24% dietary lipid groups. The liver, as the hub of lipid metabolism and the primary site for lipolysis and lipogenesis (45), is highly responsive to dietary lipids (46). Key enzymes and transcription factors play pivotal roles in lipid production and breakdown (47). Our study identified differential expression of key enzymes in pathways such as cholesterol metabolism, fat digestion and absorption, PPAR signaling, and fatty acid degradation, reflecting liver metabolic function alterations. We focused on the trends and functions of these enzymes to understand lipid metabolism regulation. Lipase, a member of the carboxyl ester hydrolase family, hydrolyzes triglycerides (TG) into glycerol and fatty acids. Lipoprotein lipase (LPL), within this family, regulates TG breakdown (48). Fatty acid-binding proteins bind free fatty acids and other hydrophobic ligands, facilitating their transport to organelles for processes such as fatty acid oxidation and TG and phospholipid synthesis (49). This study observed significant downregulation of lipase and LPL at 24% dietary lipid, suggesting inhibited lipoprotein hydrolysis and lipid absorption, and a reduction in fatty acid-binding protein levels, indicating decreased TG synthesis. This gene expression differentiation and metabolic pathway alteration might result from a negative feedback mechanism, preventing cytotoxicity from excessive lipid accumulation (50). Once ample lipid is stored in Amur grayling’s liver or other tissues in high-lipid diet groups, lipid intake and synthesis are curbed via metabolic pathways. These findings align with studies on large yellow croaker, where high lipid levels (18%) led to reduced LPL and related gene expressions (19). Intriguingly, the Amur grayling liver also appeared to engage a positive feedback mechanism to metabolize excess lipids, especially TG. Fatty acid β-oxidation, crucial for maintaining liver TG balance, involves CPT1 as a rate-limiting enzyme (51). The transcription level of CPT1 increased in the liver at 24% lipid, potentially counteracting oxidative stress and liver damage from lipid accumulation through β-oxidation, preventing lipid droplet accumulation and lipotoxicity (52). This indicates Amur grayling’s attempt to balance lipid intake and breakdown, although the equilibrium was not fully achieved. Future studies are warranted to further elucidate Amur grayling’s lipid metabolism regulation through genetic or nutritional interventions.

## Conclusion

This study is the first to investigate the lipid requirements and lipid metabolism mechanisms in Amur grayling, an economically valuable species. Our findings suggest that a dietary lipid level of 18% fulfills the lipid requirements for Amur grayling, leading to enhanced growth performance, improved intestinal digestion, optimized serum lipid profile, and augmented liver antioxidant capacity. Transcriptome analysis revealed four differentially expressed genes (DEGs)-lipase, LPL, fatty acid-binding protein, and CPT1, suggesting that the Amur grayling liver employs both positive and negative regulatory mechanisms in response to excessive dietary lipid. Quadratic regression analysis determined the optimal dietary lipid levels to be 16.62 and 16.52%, based on WGR and FCR, respectively. These outcomes offer valuable theoretical insights for developing specialized feeds for Amur grayling and provide an in-depth understanding of its lipid metabolism mechanisms.

## Limitations and prospects

First, the observed growth performance in our feeding trial highlighted a significant issue of slow growth in Amur grayling fry, attributable to factors such as breeding environment, feed composition, and feeding practices. We believe the most critical factor is the lack of comprehensive understanding of Amur grayling’s nutritional requirements. Enhancing growth rates through nutritional interventions is poised to become a focal point of future research.

Verifying gene expression using qPCR is crucial for elucidating Amur grayling’s adaptive mechanisms to dietary lipid nutrition. However, this study faced two main limitations. First, the scarcity of research on Amur grayling has resulted in limited availability of related gene sequences, an issue we aim to address in future studies. Second, given this constraint, we conducted liver transcriptome analysis using Eukaryotic reference-free transcriptome cloud analysis. This approach underscores our study’s objective to uncover the liver’s key adaptation mechanisms to dietary lipids and the genes involved in essential signaling pathways, utilizing cutting-edge transcriptome technology. This lays the groundwork for cloning-related genes in future investigations.

## Data availability statement

The raw data supporting the conclusions of this article will be made available by the authors, without undue reservation. The data presented in the study are deposited in the National Center for Biotechnology Information (NCBI) Sequence Read Archive (SRA) database, accession number PRJNA1089361. This data can be found in http://www.ncbi.nlm.nih.gov/bioproject/1089361.

## Ethics statement

The animal studies were approved by the Committee for the Welfare and Ethics of Laboratory Animals of Heilongjiang River Fisheries Research Institute (CAFS). The studies were conducted in accordance with the local legislation and institutional requirements. Written informed consent was obtained from the owners for the participation of their animals in this study.

## Author contributions

ZF: Writing – original draft, Project administration, Funding acquisition. KM: Methodology, Writing – review & editing. YW: Writing – review & editing, Formal analysis, Data curation. LW: Conceptualization, Supervision, Writing – review & editing, Resources. YZ: Writing – review & editing, Funding acquisition, Project administration. CL: Writing – review & editing, Investigation. JiaL: Writing – review & editing, Formal analysis, Data curation. DW: Resources, Writing – original draft. JinL: Resources, Writing – original draft. ZL: Investigation, Writing – review & editing.

## Footnotes

^1^ https://github.com/jstjohn/SeqPrep

^2^ https://github.com/najoshi/sickle

^3^ http://trinityrnaseq.sourceforge.net/
